# Mapping for Health in Cameroon: Polio Legacy and Beyond

**DOI:** 10.1093/infdis/jix008

**Published:** 2017-07-01

**Authors:** Louie C. Rosencrans, Gerald E. Sume, Jean-Christian Kouontchou, Arend Voorman, Yaw Anokwa, Maurice Fezeu, Vincent Y. Seaman

**Affiliations:** 1 Global Immunization Division, Centers for Disease Control and Prevention, Atlanta, Georgia;; 2 World Health Organization Country Office for Cameroon, Yaoundé;; 3 Bill and Melinda Gates Foundation, and; 4 Nafundi, Seattle, Washington; and; 5 Health Information Unit, Cameroon Ministry of Public Health, Yaoundé

**Keywords:** Cameroon, EPI, poliomyelitis, GIS, mapping, smartphones.

## Abstract

During the poliovirus outbreak in Cameroon from October 2013 to April 2015, the Ministry of Public Health’s Expanded Program on Immunization requested technical support to improve mapping of health district boundaries and health facility locations for more effective planning and analysis of polio program data. In December 2015, teams collected data on settlements, health facilities, and other features using smartphones. These data, combined with high-resolution satellite imagery, were used to create new health area and health district boundaries, providing the most accurate health sector administrative boundaries to date for Cameroon. The new maps are useful to and used by the polio program as well as other public health programs within Cameroon such as the District Health Information System and the Emergency Operations Center, demonstrating the value of the Global Polio Eradication Initiative’s legacy.

Poliomyelitis is a paralytic disease which was targeted for eradication by the 1988 World Health Assembly. There have been great strides made toward polio eradication in sub-Saharan Africa, with cases of wild poliovirus detected only in northern Nigeria between August 2014 and August 2016 [1, 2].

Cameroon is important to the historical epidemiology of infectious diseases in West and Central Africa, bordering 6 countries in the region, including Nigeria, which is currently experiencing a resurgence in circulation of WPV1 [1]. Cameroon has had a history of several polio outbreaks following importation from neighboring countries, such as from 2003 to 2009 [3]. After an importation from Chad, Cameroon experienced an outbreak of wild poliovirus type 1 (WPV1) with 9 laboratory-confirmed cases detected from October 2013 to July 2014 [2]; the outbreak was officially declared over in April 2015 [4]. In April 2014 the poliovirus outbreak spread from Cameroon into Equatorial Guinea [5]. Later international spread of WPV was declared a Public Health Emergency of International Concern, and Cameroon was classified as an exporter of wild poliovirus [6, 7]. As such, the Global Polio Eradication Initiative (GPEI) recommended “extraordinary measures” to prevent continued international spread [8].

To safeguard polio elimination in the country and prevent poliovirus spread in the region, the Expanded Program on Immunization (EPI) must maintain high-quality polio surveillance and vaccination programs. Program performance is monitored using acute flaccid paralysis (AFP) surveillance data, routine immunization coverage data, and polio mass campaign quality data, among other sources. Accurate maps are required to visualize the geographic context of these data, which are crucial for both program planning and evaluation. Accurate administrative boundaries are also needed to correctly attribute georeferenced data to administrative areas. This is important as cellphones equipped with the Global Positioning System (GPS) are increasingly being used by health programs to collect georeferenced public health data.

Another use of base maps is for microplanning. Microplans are documents which list all settlements, including total population and target population per settlement; the areas which are difficult to access and the reasons for inaccessibility; and the assets and resources needed to conduct high-quality immunization campaigns [9]. Most microplans in the African region, and until recently in Cameroon, used hand-drawn maps which were not geographically accurate [10].

In 2014, during the polio outbreak in Cameroon, there was an urgent need to conduct epidemiological analysis of AFP surveillance and routine immunization coverage data at the health district and health area levels. Unfortunately, the health district boundary map available at the time was marginally useful, showing only 179 of the 189 health districts, because of the creation of new health districts over time. Furthermore, the existing health district boundaries were also inaccurately delineated by multiple agencies over time.

After the success of detailed mapping efforts by the polio program in Nigeria [10], the EPI program of the Cameroon Ministry of Public Health (MOPH) requested technical support from the US Centers for Disease Control and Prevention (CDC) and the World Health Organization (WHO) Cameroon country office to update the maps and shapefiles using geographic information systems (GIS) to reflect all 189 health districts, as well as to create for the first time a map showing the boundaries of all 1798 health areas, the administrative level below health districts. The MOPH also requested to include locations of health facilities, schools, churches, mosques, and markets to facilitate polio immunization campaigns and other health interventions that make use of these sites.

Importantly, nonpolio programs will also be able to access and use these GIS base maps for their own epidemiological data, illustrating the broader, lasting benefits from the polio eradication program. Funding and technical support was provided by the Bill and Melinda Gates Foundation (BMGF), with further technical support from the WHO Cameroon country office, CDC, Nafundi, eHealth Africa (Nigeria), and Novel-T (Geneva).

## METHODS

### Satellite Imagery and Feature Extraction

High-resolution satellite imagery (Advanced Country Coverage, OR2A, 50 cm panchromatic) was procured from DigitalGlobe as a mosaicked (cached) file. The raw imagery (8 band, panchromatic) was sent to the Geographic Information Science and Technology (GIST) Team at Oak Ridge National Laboratories for the extraction of building and settlement features using a semiautomated algorithm. This feature extraction layer was used to identify settlement locations in 26 health districts considered to be of high programmatic priority because of the risk for polio transmission. Unfortunately, <20% of the feature extraction was processed in time for the initial mapping effort described herein.

A GIS expert was hired in-country to provide technical expertise, along with 13 other cartographers under his supervision. The MOPH Health Information Unit (HIU) was selected as the office to conduct the activity, host the cartographers, and maintain the collected data, in part because the GIS mapping results were to be integrated into the District Health Information System (DHIS2) hosted by the HIU.

The GIS mapping in Cameroon was inspired by a similar project in neighboring Nigeria. In 2013–2014, Nigeria’s polio program produced highly detailed GIS maps to support polio microplanning and vaccination team tracking [10]. In early October 2015, the data manager from the WHO Cameroon country office EPI program visited the eHealth Africa offices in Kano, Nigeria, to learn from their experiences and apply those experiences in the Cameroon context. Novel-T also provided remote technical support from Geneva, Switzerland, when there were technical issues related to the smartphones’ connectivity to satellites.

### Data Collection

Approximately 495 GPS-enabled Huawei Y320 smartphones were donated by BMGF to be used for data collection. The use of smartphones for public health data collection has been well documented as an affordable and useful tool for public health programs in developing countries [11]. In November 2015, technical consultants from CDC, BMGF, and Nafundi provided technical support for the pilot project and programmed the smartphones. The Nafundi technical expert taught the core mapping team (MOPH, WHO, CDC, BMGF, Nafundi, and the GIS expert) how to use Open Data Kit (ODK) which is a free, open-source mobile data collection software. Specifically, the Nafundi consultant taught how to design a form, how to validate and send the form to a server, how to download the forms to smartphones, and how to use the ODK Collect smartphone application.

**Figure 1.**
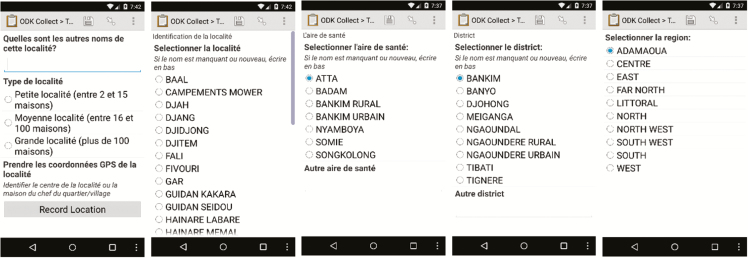
Example of Open Data Kit (ODK) data entry screens used by data collection teams.

A data collection process was created by the mapping group, to be used by teams in the field to collect GPS locations of points of interest (eg, boundaries, health facilities, settlements, markets, churches and mosques, schools) along with relevant metadata. The process was then coded as an ODK form (Figure 1) that would run on smartphones. A list of health districts, areas, settlements, neighborhoods, and points of interest from the microplans were used to populate a drop-down list for data entry on the smartphones. The primary goal of this project was to map these known features in the microplans of each health area. However, surveyors were also able to add additional points of interest if not on this list. Collected data were sent to a cloud-based ODK Aggregate server. Standard operating procedures were developed by the core technical mapping team for the GPS teams to conduct the activity.

A pilot project was conducted over 2 days in November 2015 to map Soa Health District of Centre region. Soa Health District was selected for the pilot due to its mix of urban and rural areas and its proximity to Yaoundé, the capital of Cameroon. In addition, Soa was one of the few health districts in Cameroon whose health areas had been digitally mapped in the past, and thus provided reference and validation for the new procedure. Because of a delay in the automated feature extraction, manual feature extraction was conducted to mark all settlements to be visited by the data collection teams in Soa Health District. Teams were sent with a local guide to each health area within Soa Health District, and the guides directed the teams to all settlements and other points of interest.

The main data collection activity was conducted in December 2015. A training of trainers was conducted in Yaoundé by staff from MOPH-HIU, EPI, WHO, BMGF, Nafundi, and CDC; the trainees included the 13 local GIS experts and staff from each of the 10 regional MOPH offices. After the training, technical experts including CDC, WHO, and MOPH staff were sent to each of Cameroon’s 10 regions to conduct the 2-day regional-level training for local staff and supervise the data collection activities. Local field activities were planned in part using available satellite imagery. Local MOPH staff were trained on the use of the standard operating procedures and smartphones for data collection.

The data collection field work was conducted during 15–20 December 2015. Each of the 189 health districts was visited by a team of 2 surveyors comprised of a government health system employee and a data collector. In each health area, teams were accompanied by a local guide with strong knowledge of locations of settlements, health facilities, and other points of interest. The data were automatically uploaded to the server, as soon as the surveyor was in an area with cellphone network or Wi-Fi reception.

During this process the WHO data manager in Yaoundé monitored the submitted data in real time each day of the activity and provided timely feedback to surveyors who were having technical issues. In addition, the HIU distributed a hard-copy questionnaire to the health facilities visited by the data collection teams to collect more detailed information data for entry into the DHIS2 system.

### GIS Map Construction

Data consolidation and cleaning were conducted by GIS technicians and WHO and MOPH EPI data management staff during January and February 2016, and then the GIS technicians at HIU determined the borders of health areas based on GPS points and local knowledge of the guides from the December data collection. Thus, if the guide had indicated the location of a health area border, the data collection team would take the coordinates and any relevant notes regarding the border type (such as being marked by a river, road, or other type of feature). If a river or a road was listed as the border for a certain section of the health area boundary, then the GPS points were taken by the data collection team. Subsequently, those features were traced from the satellite imagery using ArcGIS software by the GIS consultants and were used to delineate the boundary shapefile.

## RESULTS

**Figure 2.**
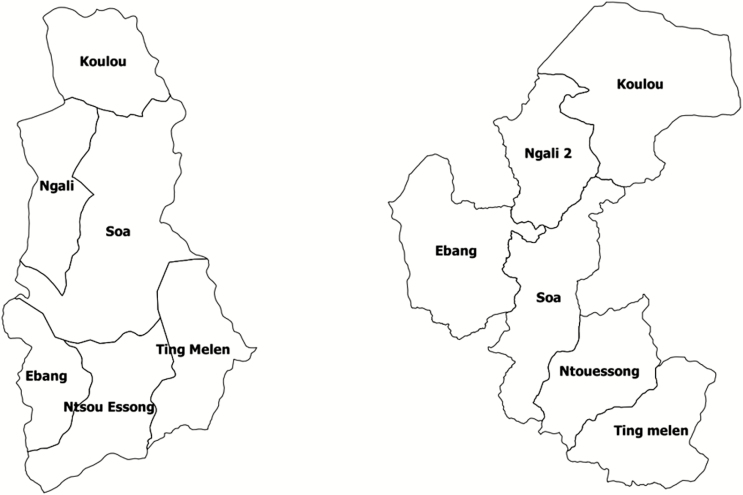
Soa health district health area boundaries, before (left) and after (right) pilot.

The pilot activity in Soa Health District was completed in 2 days, and the results showed that the older map had been inaccurate with significant differences between the old and new health area boundaries, as seen in Figure 2. This illustrated the potential for nationwide improvement of border accuracy through the process of data collection and redigitizing borders.

A total of 77778 points of interest were visited from the 15 to 20 December 2015, and data with GPS coordinates were collected. After deleting incomplete or inaccurate data in Yaoundé, 75809 were used in the map-making process. These included 20741 settlements; 19564 churches and mosques; 18086 schools (nursery, primary, secondary, and university); 4775 health facilities (public, private, nongovernmental organization, religion-affiliated); 3037 markets; and 9606 health area boundary points.

Compared to the microplan reference, 76.7% of settlements were visited by the survey teams, as well as 65.2% of markets, 70.1% of schools, and 72.8% of churches and mosques. Some missed settlements are in areas of Cameroon difficult to access, for example, in the Far North Region with security challenges. On the other hand, more health facilities were visited than were registered; these extra facilities were comprised of unofficial or unregistered health facilities, pharmacies, and private laboratories.

**Figure 3.**
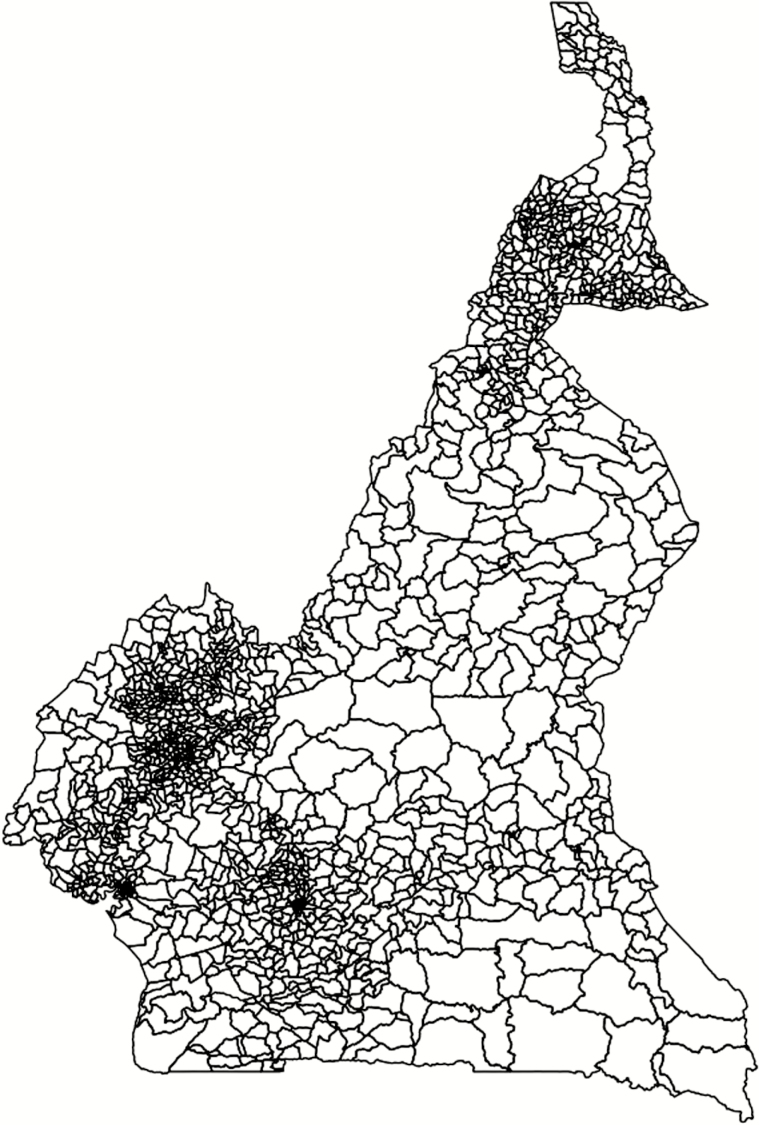
Cameroon health area boundaries.

Using the methods described above, borders were drawn (or redrawn if any digitized health area borders already existed) for all 1798 health areas (Figure 3).

**Figure 4.**
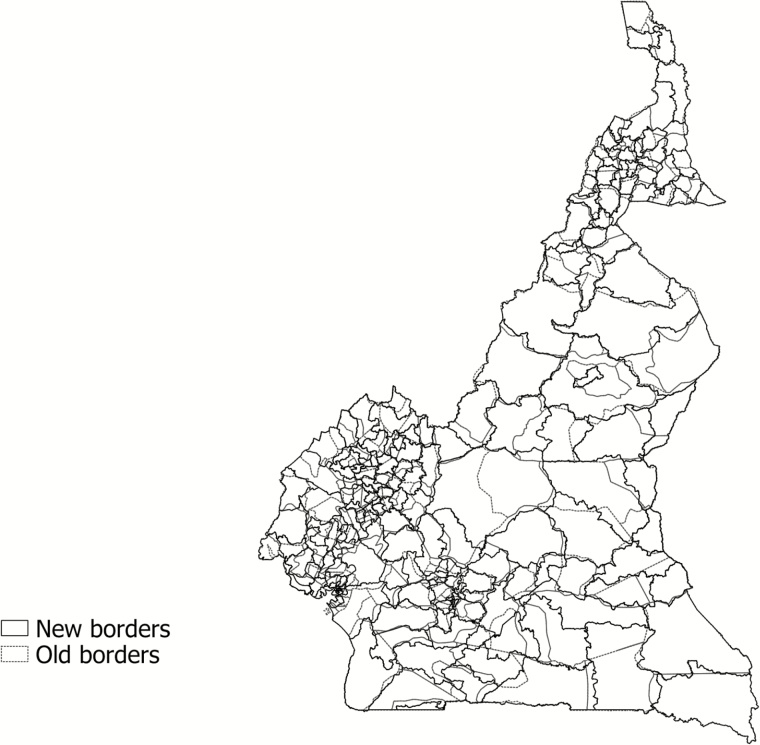
Cameroon health district borders.

Figure 4 compares the old and new Cameroon health district borders. Distances between the old and new borders are as large as 101 km as seen in Yoko health district in Centre Region. There was a mean absolute change of 96% in the area in square kilometers between the old and new health districts. Health districts with a change in area of 200% or more were all because of reductions, with the largest being in Logbaba health district of Littoral region, which went from 289 km^2^ in the old health district map to 13 km^2^ in the new map.

**Figure 5.**
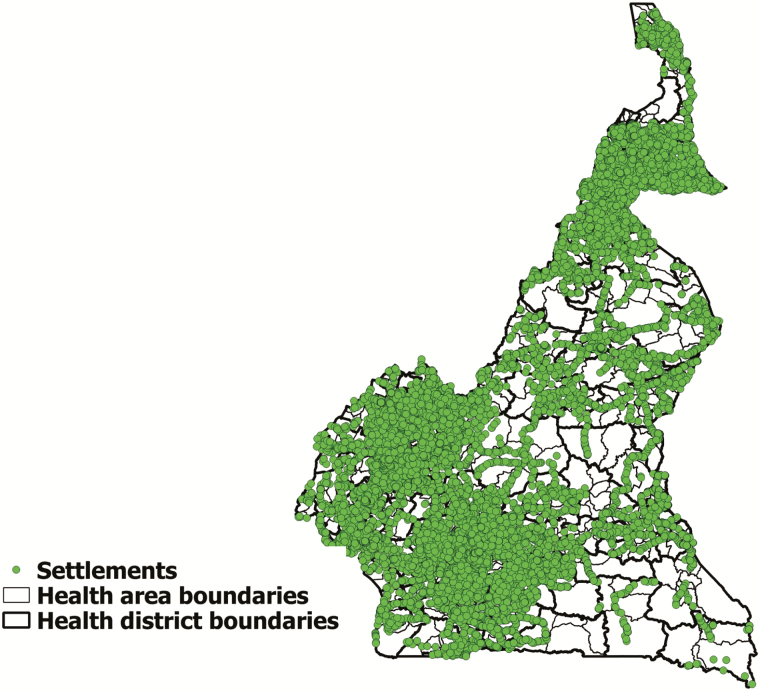
Settlements located by data collection teams during 15–20 December 2015.

As seen in Figure 5, there are areas of Cameroon such as in the Far North in which accessibility was difficult, while in other areas there was a relatively low density of settlements.

**Figure 6.**
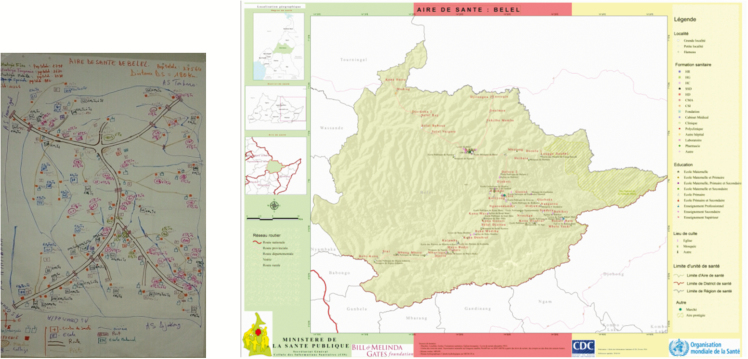
Belel health area microplan maps, old (left) and new (right).

An example of the old hand-drawn microplan maps and new digitally generated map can be seen in Figure 6, showing Belel Health Area of Ngaoundéré Rural Health District. With the new map district and health area, managers will have more accurate tools for planning polio immunization campaigns and other public health activities.

After the creation of the new maps, a cascade training on their use was conducted, starting at the national level in March 2016. The training focused on mapping immunization data (ie, polio surveillance data, routine immunization coverage data, postimmunization campaign survey data) using Quantum GIS version 2.12 (QGIS).

### Discussion

In this article, we described how the GPEI partners and the government of Cameroon collaborated to create detailed health area and health district maps for use in polio program planning, monitoring, and evaluation, as well as for use by nonpolio health programs. We used satellite imagery and GPS-enabled smartphones to delineate the 189 health districts and 1798 health areas used by the polio program, in addition to >75000 geographic points of interest. We found that the new health district maps differed substantially from those used previously.

The WHO and MOPH are now using smartphones with ODK mobile data collection software for several purposes, including conducting postvaccination campaign quality assessment surveys, AFP case investigations, supportive supervision, and active surveillance visits to health facilities. These and other health staff activities are now geolocated to ensure that human resources are being deployed in the expected geographic areas.

An important benefit of the upgraded GIS maps is their use by other disease control programs within the Cameroon Ministry of Public Health. The EPI routine immunization program used the maps and smartphones to monitor the April 2016 switch from trivalent to bivalent oral polio vaccine. The maps are now based in the HIU, which is responsible for hosting and maintaining the DHIS2, processing health statistics, and publishing and disseminating health data. DHIS2 contains data from various programs such as Integrated Disease Surveillance and Response (IDSR), EPI, Maternal Health, and Malaria [12]. IDSR and other disease control programs will be able to use the GIS maps, increasing their analytic capability (O. Pasi, personal communication).

The Cameroon MOPH’s newly formed Emergency Operations Center (EOC) is planning to incorporate these GIS maps for use in outbreak response. The EOC is housed within the Directorate for the Fight Against Disease, Epidemics and Pandemics, and data from DHIS2 will feed into an epidemiological data dashboard at the EOC (O. Pasi, personal communication). Use of the national GIS maps will greatly enhance the ability of the EOC to analyze data and respond to outbreaks.

There is now a team of MOPH staff who have been trained on the use of GIS software for epidemiological analysis of program data. The HIU has uploaded new map images in PDF format from this mapping project at region, health district, and health area levels to the Cameroon MOPH website (https://www.dhis-minsante-cm.org/portal/).

Future work will identify remaining missed settlements through comparison with the villages identified through feature extraction to determine which settlements had been missed during the December 2015 fieldwork. These settlements can then be targeted for visits by GPS teams, which will also visit and collect data on previously missed health facilities, schools, churches and mosques, and markets. Missed settlements can also be visited and GPS coordinates attained during visits by other means such as district or health area health staff visiting to conduct postimmunization campaign surveys, or case investigations, as those activities are now conducted using GPS-enabled smartphones. In addition, since the maps were distributed nationwide, feedback has been gathered from each of the health areas regarding the new maps. For example, personnel from a few health areas and health districts have noted that the boundaries are in need of further modification, and this feedback will be incorporated into the next phase of map editing.

This work shows how the technology and lessons learned from polio eradication activities in Nigeria can be rapidly applied and scaled up in other contexts. In Cameroon, field activities took only 6 days, followed by only 2 months of desk work to prepare maps. This relatively short exercise provides a valuable resource for both the polio program and the MOPH of Cameroon. This experience also shows how strong leadership and commitment from the MOPH can yield high-impact results in a context of limited financial resources.

The existence of accurately geolocated features allows for stronger program coordination and evaluation in Cameroon by public health programs. While other disease control programs in Cameroon have used GIS and smartphones in the past, the expanded use of these tools for polio eradication has reinforced and increased the capability of the Cameroon MOPH to use these important tools for other disease control programs. The improved GIS maps and the expansion of the use of smartphones with data collection capability throughout the MOPH and WHO is an important example of the legacy of polio.

## Notes

***Acknowledgments.*** We thank the Cameroon Ministry of Public Health, the Cameroon WHO Office, the MOPH Health Information Unit, the GIS technician Joachim Etouna, GIS consultants, and the hundreds of data collectors and guides who made this project possible.

***Financial support.*** Funding for this project was provided by the Bill and Melinda Gates Foundation.

***Supplement sponsorship.*** This work is part of a supplement coordinated by the Task Force for Global Health with funding provided by The Bill and Melinda Gates Foundation and the Centers for Disease Control and Prevention.

***Potential conflicts of interest.*** All authors: No reported conflicts. All authors have submitted the ICMJE Form for Disclosure of Potential Conflicts of Interest. Conflicts that the editors consider relevant to the content of the manuscript have been disclosed.
